# Working Memory Recovery in Adolescents with Concussion: Longitudinal fMRI Study

**DOI:** 10.3390/jcm13123585

**Published:** 2024-06-19

**Authors:** Anna Manelis, João P. Lima Santos, Stephen J. Suss, Cynthia L. Holland, Courtney A. Perry, Robert W. Hickey, Michael W. Collins, Anthony P. Kontos, Amelia Versace

**Affiliations:** 1 Department of Psychiatry, University of Pittsburgh, Pittsburgh, PA 15213, USA; limasantosjp2@upmc.edu (J.P.L.S.); ssuss007@fiu.edu (S.J.S.); 2 Department of Orthopaedic Surgery/UPMC Sports Medicine Concussion Program, University of Pittsburgh, Pittsburgh, PA 15213, USA; clh197@pitt.edu (C.L.H.); cap236@pitt.edu (C.A.P.); collinsmw@upmc.edu (M.W.C.); kontosap@upmc.edu (A.P.K.); 3 Department of Pediatric Emergency Medicine, Children’s Hospital of Pittsburgh, University of Pittsburgh Medical Center, University of Pittsburgh, Pittsburgh, PA 15224, USA; robert.hickey@chp.edu; 4 Department of Radiology, Magnetic Resonance Research Center, University of Pittsburgh Medical Center, University of Pittsburgh, Pittsburgh, PA 15213, USA

**Keywords:** concussion, adolescents, working memory, fMRI, recovery

## Abstract

**Background**: Understanding the behavioral and neural underpinnings of the post-concussion recovery of working memory function is critically important for improving clinical outcomes and adequately planning return-to-activity decisions. Previous studies provided inconsistent results due to small sample sizes and the use of a mixed population of participants who were at different post-injury time points. We aimed to examine working memory recovery during the first 6 months post-concussion in adolescents. **Methods**: We used functional magnetic resonance imaging (fMRI) to scan 45 concussed adolescents [CONCs] at baseline (<10 days post-concussion) and at 6 months post-concussion. Healthy control adolescents [HCs; n = 32] without a history of concussion were scanned once. During the scans, participants performed one-back and two-back working memory tasks with letters as the stimuli and angry, happy, neutral, and sad faces as distractors. **Results**: All affected adolescents were asymptomatic and cleared to return to activity 6 months after concussion. Working memory recovery was associated with faster and more accurate responses at 6 months vs. baseline (*p*-values < 0.05). It was also characterized by significant difficulty-related activation increases in the left inferior frontal gyrus (LIFG) and the left orbitofrontal cortex (LOFC) at 6 months vs. baseline. Although the activation differences between one-back and two-back were comparable between HCs and CONCs at 6 months, HCs had more pronounced activation in the LIFG than concussed adolescents. **Conclusions**: Post-concussion recovery is associated with significant performance improvements in speed and accuracy, as well as the normalization of brain responses in the LIFG and LOFC during the n-back task. The observed patterns of LOFC activation might reflect compensatory strategies to distribute neural processing and reduce neural fatigue post-concussion.

## 1. Introduction

The lifetime prevalence of concussion among adolescents is rising and increased from 19.5% in 2016 to 24.6% in 2020 [1]. Post-concussion recovery usually takes around four weeks, but in some cases may take longer [2]. Longitudinal studies comparing cognitive and brain function changes during recovery may shed light on the mechanisms underlying post-concussion impairments and recovery.

Post-concussion symptoms in adolescents impact their cognitive and social function [3,4] in addition to eliciting movement-related dizziness, nausea, vision alteration, excessive fatigue, and other physiological symptoms [5,6,7]. Working memory, which is the ability to update and manipulate information online [8,9], is one of the cognitive domains frequently affected by concussion [10,11]. Performance on working memory tasks relies on the prefrontal, cingulate, and posterior parietal cortical regions that increase in activation when the task becomes more difficult [8,12,13,14,15,16]. These regions are actively developing in adolescents [17,18,19,20], so if concussion affects these regions, the development of cognitive function may also be impacted.

Working memory is often assessed using n-back tasks in which participants are presented with a sequence of stimuli and have to indicate whether the current stimulus is the same as the stimulus presented one or more items before [12,15,16]. Previous cross-sectional neuroimaging studies have reported inconsistent findings regarding the impact of concussion on working memory task performance and corresponding brain activation patterns. For example, several studies found no differences in behavioral performance on working memory tasks between concussed and control individuals at 1 month [21], 7.5 months [22], or 2.5 years [23] post-concussion. However, other studies found reduced working memory task performance in the acute/subacute post-concussion period [16,24] as well as three months after injury [25]. Working memory task performance was associated with lower prefrontal cortical and parietal activation [24,25] in concussed vs. control adolescents. More symptomatic participants showed greater differences in retrosplenial cortex activation for the one-back vs. two-back condition than their less symptomatic counterparts within the first 10 days of injury [16]. Brain activation in the temporal and frontal brain regions was greater in concussed adolescents during the two-back condition at 7.5 months [22]. There were no significant differences in working memory-related brain activation between concussed and control adolescents 2.5 years after injury [23].

The findings cited above suggest that cross-sectional studies are not ideal for capturing changes across post-concussion recovery. Therefore, conducting longitudinal studies that compare working memory performance immediately after concussion with that after full clinical recovery is critically important. Only a small number of studies have examined post-concussion recovery longitudinally. One study scanned youth athletes three times: within 72 h, at 2 weeks, and 2 months post-concussion [26]. Despite comparable behavioral performance at all time points, Detwiller et al. reported greater activation in bilateral dorsolateral prefrontal and inferior parietal cortices in concussed participants relative to controls for two-back vs. one-back conditions at baseline and two weeks post-concussion [26]. The other study scanned adults one month after a concussion and six weeks after the first scan. They found that affected individuals were not able to adequately increase their brain activation for difficult vs. easy working memory tasks one month after injury. This ability, however, improved at the six-week follow-up [27]. A third study examined behavioral and brain alterations after one month of post-concussion recovery and found that performance on a difficult working memory task became more accurate and elicited greater prefrontal activation in concussed adolescents [11].

The inconsistent results reported by the previous cross-sectional and longitudinal studies could be explained by small sample sizes and the inclusion of a mixed population of participants who were not fully recovered with those who were recovered after concussion. The goal of our study was to examine the post-concussion recovery of working memory in affected adolescents by comparing their behavioral performance and brain activation during the acute/subacute period (<10 days after concussion) with that at six months after injury. Based on the extant literature briefly reviewed above, we hypothesized that the post-concussion recovery of working memory would be characterized by activation changes in the prefrontal, parietal, and PCC regions during the performance of difficult vs. easy working memory tasks. We also hypothesized that behavioral performance (i.e., accuracy and reaction time (RT)) would improve across recovery and become similar to that of HCs.

## 2. Materials and Methods

### 2.1. Participants

The study was approved by the University of Pittsburgh Institutional Review Board (IRB number STUDY19030360). Written informed assent was obtained from all participants, and written informed consent was obtained from all caregivers. All participants were adolescents between 12 and 17 years of age. Adolescents with concussion (CONCs) were diagnosed using current consensus criteria [28] within 1–10 days after injury. Those who lost consciousness for more than 5 min at the time of the injury were excluded from the study. Healthy control adolescents (HCs) had no history of concussion and were age- and sex-matched to those with concussion. Exclusion criteria for all adolescents included an IQ below 70; MRI contraindications; systemic medical, neurologic, psychotic spectrum, and neurodevelopmental disorders; orthopedic injury within the past month; alcohol or illicit substance use/dependency within the past three months, intoxication, or presence of illicit drugs in a urine test on the day of the scan; and left/mixed handedness.

CONCs were scanned twice—at baseline during the subacute phase of concussion recovery (1–10 days after concussion) and 6 months after concussion. Only the individuals who attended both scans and had usable data were included in the longitudinal data analysis. Due to budgetary limitations, the HCs were scanned one time. A total of 77 adolescents (45 CONCs and 32 HCs) met eligibility criteria, had usable fMRI data, and therefore, were included in the behavioral and neuroimaging data analyses. Of these individuals, cross-sectional findings have already been reported for 26 CONCs and 27 HCs [16].

### 2.2. Clinical Assessments

The presence of psychiatric disorders and/or current use of psychotropic medications was examined using the International Neuropsychiatric Interview for children and adolescents (MINI-KID) [29]. Concussion symptoms were measured using the Post-Concussion Symptom Scale (PCSS) [30] and Vestibular/Ocular-Motor Screening (VOMS) [6] that were administered by a trained clinician. Higher scores on all three assessments indicated greater severity. Considering that in our previous cross-sectional study of subacute concussion, only the VOMS scores were associated with the changes in brain activation during the n-back task, we used this measure as a covariate in the behavioral neuroimaging data analysis in this longitudinal study. All relevant demographics and medical history, as well as the mechanism of concussion, are reported in Table 1.

### 2.3. Experimental Paradigm

**Figure 1:**
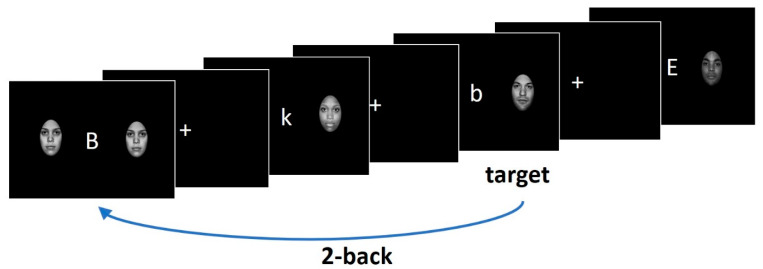
The n-back task paradigm (the figure is adopted from [16]).

The details of the n-back task used in this study to measure working memory are described elsewhere [16]. In short, participants were presented with lowercase and capital letters one at a time. In the 1-back condition, they had to indicate whether the letter presented on the screen matched the one shown in the previous trial. In the 2-back condition, participants had to indicate whether the letter presented on the screen matched the letter presented two trials ago. In each trial, the letters were presented with either angry, happy, neutral, or sad faces located on the right and left sides of the letter (Figure 1). The faces were taken from the NimStim database [31] and served as emotional distractors [32]. Participants performed three 5 min runs consisting of eight 30 s blocks of trials (1-back and 2-back tasks with 4 emotional face distractor conditions) presented in random order and preceded by a 4.5 s instruction screen. Participants had to respond as quickly and accurately as possible by pressing the response button with the index finger of one hand for the letters that were in the 1- or 2-back position and with the other hand for all other stimuli. We counterbalanced hand assignment across participants.

### 2.4. Behavioral Data Analysis

We used three-way mixed-effects linear models (‘lme4’ package in R [33]) with group (CONC 1, CONC 2, HC), n-back condition (1- and 2-back), and distractor emotions (happy, angry, sad, neutral) as independent variables to explain reaction time (RT) and accuracy (in the separate models) using Type III Analysis of Variance with Satterthwaite’s method. Participants were treated as a random factor, and their age, sex, and VOMS scores at baseline served as covariates. When appropriate, the contrasts and means were estimated from the mixed-effects models (‘modebased’ package in R [34])). Tukey’s Honestly Significant Difference (HSD) was used to correct for multiple comparisons.

### 2.5. fMRI Data Acquisition

Functional MRI data were acquired at the University of Pittsburgh using a Siemens PRISMA 3T MR system and a 32-channel RF head coil. The MPRAGE sequence was used to acquire a high-resolution structural image (voxel size = 1 × 1 × 1 mm^3^, TR = 2400 ms, FOV = 256 mm, flip angle = 8°, 176 slices). A gradient-echo, echo-planar sequence was used to collect functional MRI data (360 volumes per run; voxel size: 2 × 2 × 2 mm^3^, TR = 800 ms, echo time = 30 ms, field of view = 210 mm, flip angle = 52°, 72 slices, multiband acceleration factor = 8). Two spin echo images with the anterior-to-posterior and posterior-to-anterior phase-encoding directions (voxel size = 2 × 2 × 2 mm^3^, TR = 8000 ms, TE = 66.00 ms, FOV = 210 mm, flip angle = 90°, 72 slices) were collected to allow for the geometric distortion correction of fMRI data.

### 2.6. fMRI Data Preprocessing

DICOM files were converted to NIFTI using the dcm2niix tool (v1.0.20180826 BETA GCC5.4.0) [35]. Non-brain tissues were removed using the optiBET script [36]. Motion correction was performed using MCFLIRT [37]. Spatial smoothing with a Gaussian kernel of full-width at half-maximum = 6 mm was applied. Susceptibility distortion in the BOLD images was corrected using topup in FSL6.0.3 (www.fmrib.ox.ac.uk/fsl, accessed on 17 June 2024). BOLD images were transformed to MNI space by first registering BOLD images to the high-resolution structural (MPRAGE) images using FLIRT (FMRIB’s Linear Image Registration Tool, version 6.0) [37,38] with Boundary-Based Registration (BBR), and then registering the high-resolution images to the MNI152 T1-2mm template using FNIRT (FMRIB’s Non-linear Image Registration Tool, version 6.0) [39] with nine degrees of freedom (DOF). The two resulting transformations were concatenated and applied to the original BOLD. The quality of transformation was examined.

Motion artifacts were removed from BOLD images using ICA-AROMA [40]. High-resolution structural images were segmented to white matter, gray matter, and cerebral–spinal fluid (CSF) using the fsl_anat script (http://fsl.fmrib.ox.ac.uk/fsl/fslwiki/fsl_anat, accessed on 17 June 2024), and the time courses associated with the white matter and CSF masks were extracted from the preprocessed functional data and regressed out during the analysis. After that, a high-pass filter (Gaussian-weighted least-squares straight line fitting, with sigma = 56.25) was applied.

### 2.7. fMRI Data Subject-Level and Group-Level Analysis

#### 2.7.1. Subject-Level Analysis

A subject-level General Linear Model analysis was implemented using FEAT (FMRI Expert Analysis Tool, v6.0). A hemodynamic response function was modeled using a Gamma function. The model included regressors for instruction screens informing about 1- and 2-back blocks as well as for 1-back and 2-back blocks with happy, angry, scared and sad face distractors. The contrasts of interest included computing the 1-back vs. 2-back differences in brain activation across all face distractors as well as for each face distractor separately. The data from the available runs were averaged for each subject and session.

#### 2.7.2. Group-Level Analysis

The first group-level analysis included the CONC group only and compared the 1-back vs. 2-back differences in brain activation at baseline (post-concussion subacute phase) vs. the 1-back vs. 2-back differences at 6-month follow-up. This longitudinal analysis was conducted using the Sandwich Estimator (swe) approach [41] for nonparametric permutation inference conducted in the whole brain with Threshold-Free Cluster Enhancement correction (TFCE) [42], 1000 permutations, and FWE-corrected *p*-values < 0.05. The *p*-values were corrected for the 2 contrasts of interest (1-back > 2-back and 2-back > 1-back) using the Bonferroni method (*p* = 0.05/2 = 0.025). Given that our previous cross-sectional study of concussed adolescents did not reveal the effect of distractor emotions on RT and accuracy [16], we decided to compare the two time points across all emotional distractors if the behavioral data analysis did not reveal the effect of emotions again. In contrast, we would examine the 2 timepoint x 4 emotions model if there was an effect of emotions on RT and accuracy.

The follow-up analyses were conducted based on the percent signal changes extracted from the brain regions showing significant differences between subacute and 6-month scans. We used mixed-effects models (as described for the analysis of behavioral data) to compare brain activation in HCs and CONCs at two time points and to understand the relationship between brain activation and behavioral measures of working memory. When appropriate, the contrasts and means were estimated from the mixed-effects models (‘modebased’ package in R [34]. Tukey’s Honestly Significant Difference (HSD) was used to correct for multiple comparisons.

## 3. Results

Unusable runs were excluded from both brain and behavior analyses. A total of 11 runs from sessions 1 and 2 were excluded. While the longitudinal analyses included only the CONC group (the HC group was scanned only once), we used the behavioral and fMRI data (extracted from the regions showing the changes in activation from baseline to 6 months) for HCs during session 1 as a comparison.

### 3.1. Demographics and Behavioral

The CONC and HC groups did not differ in age, race, and biological sex profiles (Table 1). All adolescents with concussion were cleared to return to their activities at the 6-month scan (mean number of days between injury and clearance = 30.2 days, SD = 33.9, range = 2–175 days). There were on average 159.9 days (SD = 40, range = 25–200 days) between the date of clearance for activities and the follow-up scan at 6 months.

**Figure 2:**
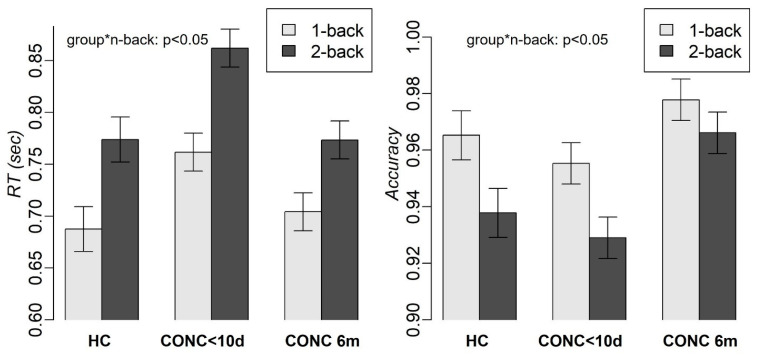
RT and accuracy in HC and adolescents with concussion during the first 10 days (CONC<10d) and 6 months (CONC 6m) after concussion. “group*n-back” refers to the interaction between group and n-back.

The mixed-effects analysis with group, n-back condition, and distractor emotions as independent variables revealed a significant group-by-n-back interaction effect (F(2, 877.19) = 3.3, *p* = 0.04) and the main effects of the n-back tasks (F(1, 877.1) = 265, *p* < 0.001) and group (F(2, 134.96) = 74.9, *p* < 0.001) on RT (Figure 2). A slower RT was observed for two-back vs. one-back (t(895.02) = 16.33, *p* < 0.001) and for subacute CONCs compared to both HCs (one-back: t(84.28) = 2.6, *p* = 0.03; two-back: t(84.28) = 3.1, *p* = 0.007) and CONCs at 6 months (one-back: t(895.02) = 6.8, *p* < 0.001; two-back: t(895.02) = 10.4, *p* < 0.001). RT did not significantly differ for HC vs. CONC at 6 months. We also observed the main effects of the n-back tasks (F(1, 876.32) = 44.5, *p* < 0.001) and group (F(2, 135.88) = 31.6, *p* < 0.001) on accuracy (Figure 2). Lower accuracy was observed for the two-back vs. one-back task (t(895.11) = −6.7, *p* < 0.001) and for subacute CONCs vs. CONCs at 6 months (t(895.11) = −7.9, *p* < 0.001). No significant effect of distractor emotions (Figure S1), age, or biological sex was observed on either RT or accuracy.

### 3.2. Neuroimaging

**Figure 3:**
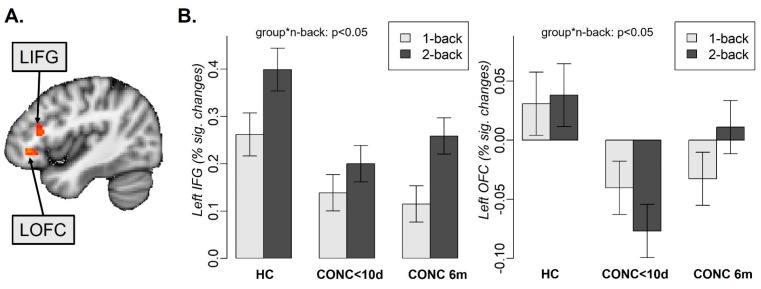
Left inferior frontal gyrus (LIFG) and left orbitofrontal (LOFC) activation during 1back and 2-back tasks in healthy controls (HC), adolescents with concussion during the first 10 days (CONC<10d) and 6 months (CONC 6m) after concussion. “group*n-back” refers to the interaction between group and n-back. (**A**) Depicts KIFG and LOFC locations, (**B**) percent signal changes in the LIFG and LOFC for 1-back and 2-back tasks.

Considering that we found no significant effect of distractor emotions on RT or accuracy, the neuroimaging data analysis was conducted across all emotions. The comparison of brain activation for two-back-minus-one-back differences for subacute vs. 6-month follow-up scans in the CONC group revealed significant longitudinal changes in the left inferior frontal gyrus (LIFG; nvox = 62, Z-max = 4.9, [−40, 24, 16]) and the left orbitofrontal cortex (LOFC, nvox = 104, Z-max = 5.5, [−38, 34, −4]) (Figure 3A).

Further analyses were conducted on the percent signal changes extracted from these regions and included the CONC group at the two time points and HCs at time point 1. To confirm that activation in the LIFG and the LOFC was not sensitive to distractor emotions, we included this variable in the mixed-effects analysis described below. The mixed-effects analysis with group, n-back condition, and distractor emotions as independent variables revealed the group-by-n-back interaction (F(2, 877.16) = 10.4, *p* < 0.001) and the main effects of the n-back tasks (F(1, 877.16) = 179.5, *p* < 0.001) and group (F(2, 134.36) = 5, *p* = 0.008) on activation in the LIFG. HCs had higher activation than subacute CONCs (t(74.47) = 2.75, *p* = 0.02) and CONCs at 6 months (t(74.47) = 2.45, *p* = 0.04). The same analysis in the LOFC revealed the group-by-n-back interaction (F(2, 876.79) = 8.5, *p* < 0.001) and the main effects of group (F(2, 135.38) = 14.3, *p* < 0.001) (Figure 3B). Subacute CONCs had lower activation than HCs (t(77.72) = −2.79, *p* = 0.02) and CONCs at 6 months (t(77.72) = −4.9, *p* < 0.001). There was no significant effect of distractor emotions (Figure S1), age, or biological sex on brain activation in either the LIFG or LOFC.

The analysis of contrasts demonstrated that concussion recovery was related to the increases in the LIFG and LOFC activation for the two-back, but not the one-back condition. Specifically, adolescents with concussion had significantly lower activation at baseline compared to at the 6-month follow-up (LIFG: t(895.01) = −4.3, *p* < 0.001; LOFC: t(895.05) = −6.4, *p* < 0.001) and compared to HCs (LIFG: t(79.45) = −3.3, *p* = 0.004; LOFC: t(94.06) = −3.3, *p* = 0.004). In the LIFG, all groups showed increased activation for the two-back vs. one-back task (HCs: t(895.01) = −8.39, subacute CONCs: t(895.01) = −4.47, CONCs at 6 months: t(895.01) = −10.43, all *p*-values < 0.001). In the LOFC, HCs showed no difference between the two- and one-back tasks (t(895.05) = 0.45, *p* = 0.66), subacute CONCs showed lower activation for the two-back vs. one-back task (t(895.05) = −2.7, *p* = 0.008), while CONCs at 6 months showed greater activation for the two-back vs. one-back task (t(895.05) = 3.2, *p* = 0.001)).

**Figure 4:**
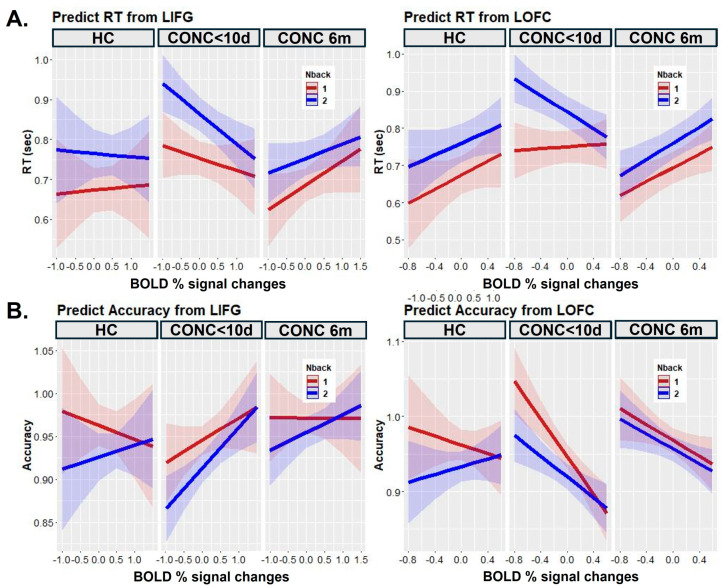
The effect of brain activation, n-back condition, and group on RT (**A**) and accuracy (**B**). “CONC<10d”—adolescents with concussion at baseline, “CONC 6m”—adolescents with concussion at 6-month follow-up. “HC”—healthy controls.

To examine the relationship between brain activation and behavioral measures of working memory, we conducted mixed-effects analysis with group, n-back condition, and brain activation as predictors and RT and accuracy as outcome variables. These analyses revealed significant group-by-LIFG (F(2, 845.98) = 10.2, *p* < 0.001) and group-by-LOFC (F(2, 925.07) = 9.5, *p* < 0.001) interaction effects on RT (Figure 4). Post hoc analysis showed that these effects were driven by brain activation in the CONC group at baseline (i.e., <10 days post-concussion)) for the two-back condition. Specifically, at baseline, the CONCs with lower activation in the LIFG (slope: t(961.06) = −3.07, *p* = 0.002) and LOFC (slope: t(911.05) = −2.97, *p* = 0.003) also had slower RTs in the two-back condition. Interestingly, at the 6-month follow-up, the same participants showed an opposite pattern of results: CONCs with lower LOFC activations showed faster RTs in the two-back condition.

There was a significant group-by-LOFC (F(2, 798.98) = 4.3, *p* < 0.05) interaction effect on accuracy. Greater activation in the LOFC corresponded to lower accuracy in the concussed, but not the control, adolescents at both the baseline and 6-month scans (slope for subacute CONCs: t(953.84) = −4.68, *p* < 0.001; slope for CONCs at 6 months: t(952.89) = −2.72, *p* = 0.007).

## 4. Discussion

Understanding the behavioral and neural underpinnings of the post-concussion recovery of working memory performance is critically important for improving clinical outcomes and informing better return-to-activity decisions. In this study, we examined how behavioral and neural correlates of working memory changed between the subacute period (<10 days) and 6 months of post-concussion recovery in affected adolescents. Consistent with multiple previous studies [12,13,14,15,16,43], participants were slower and less accurate for the more difficult two-back vs. the less difficult one-back condition. Concussed adolescents were slower and less accurate during the subacute recovery period than at 6 months after concussion, which aligned with some longitudinal studies of concussion [11] but not others [26]. During the subacute period, concussed adolescents were also slower than HCs, which parallels previous findings [16,24].

The fMRI analysis revealed that the subacute post-concussion period was characterized by aberrant brain responses in the left inferior frontal gyrus (LIFG) and orbitofrontal cortex (LOFC). These results are largely consistent with those reported previously [27]. Specifically, affected adolescents were unable to increase activation in the LIFG for the two-back vs. one-back condition immediately after concussion but were able to do so after 6 months of recovery. Moreover, the magnitude of the LIFG activation changes from the one-back to two-back condition did not differ for concussed adolescents at the 6-month follow-up and their non-concussed counterparts. The LIFG is a part of the working memory circuitry as per the NeuroSynth meta-analysis [44]. It plays a role in cognitive control, the resolution of proactive interference [45], and updating information in working memory [46]. In our study, the inability to activate the LIFG for the two-back condition was associated with a slower RT and lower accuracy in concussed adolescents in the subacute period. Our previous resting-state functional connectivity study indicated that unlike HCs showing connectivity between the IFG and dorsal attention network, concussed adolescents demonstrated no such connectivity [47]. Disconnection of the IFG from the attentional network may serve to avoid a ‘chain reaction’ and, consequently, more massive cognitive impairments when the IFG becomes dysfunctional.

The LOFC is the region located outside the working memory circuitry. The levels of activation in the LOFC were not different for the one-back and two-back conditions in HCs, thus confirming that this region is usually insensitive to working memory load. Interestingly, in concussed adolescents, activation in this region depended on the post-concussion recovery phase. While activation associated with the one-back condition remained the same, activation for the two-back condition significantly increased during 6 months of recovery. These findings may be indicative of the compensatory strategy that engages additional brain regions to perform working memory tasks after a concussion. The compensatory strategies could be related to an increase in neural effort to maintain cognitive functioning even if it is achieved at the price of a higher level of fatigue [48]. Engaging additional regions could help avoid excessive fatigue by distributing processing across more regions than is usually necessary. Considering that the lateral OFC is implicated in decision-making [49] and top–down attentional control and performance monitoring [50], engaging the LOFC later in the post-concussion recovery process might help to maintain a high level of behavioral performance. These findings suggest that the consequences of concussion on the brain may last beyond the point of return-to-play clearance, thus warranting a need for longer prospective studies.

Despite previous notions of emotional lability and reduced emotion regulation after concussion [51], we found no effect of emotional distractors on either behavioral or neural correlates of working memory in concussed adolescents neither immediately after the injury nor 6 months after that. This lack of findings suggests that concussion-related impairments in emotion processing and regulation are too subtle to be captured by fMRI.

One limitation of this study is the lack of longitudinal data for HCs, which makes it impossible to tease apart the effect of task repetition from the effect of post-concussion recovery. Adolescents with concussion were scanned twice, so their 6-month improvement in working memory might have partially been associated with task repetition. Some studies report that the task repetition effect exists even 6 months after the first exposure to the task [52]. One piece of evidence for a task repetition effect comes from the finding of higher accuracy for concussed adolescents at 6 months relative to HCs at baseline. On the other hand, all participants showed very high accuracy (>90%) in the n-back task, so the variations in the accuracy are not necessarily meaningful. Scanning HCs at a 6-month follow-up would help us understand if performing the task for the second time makes the task easier or alters brain activation for difficult vs. easy tasks. The second limitation is that this study had only two scans, which did not allow us to examine the trajectory of concussion recovery. The third limitation is the rather long period between the scans. By the 6-month follow-up, all adolescents with concussions were asymptomatic and had been cleared to return to sports and other activities. Future studies should consider scanning affected adolescents and their healthy counterparts more frequently to uncover the trajectory of post-concussion recovery. To better understand the compensatory mechanisms discussed above, future prospective studies should have a longer duration than 6 months post-injury to understand when and how brain function returns to normal.

## 5. Conclusions

In summary, our study provides insight into working memory-related behavioral and brain activation changes occurring across six months following concussion in adolescents. Participants experienced significant improvements in RT and accuracy, as well as the normalization of brain responses to the task difficulty in the LIFG and LOFC during the n-back task across a 6-month post-concussion time period. We believe that the patterns of LOFC activation reported in this study might reflect compensatory strategies to distribute neural processing and reduce neural fatigue following concussion. Considering the complexity of concussion recovery, extended prospective studies with more frequent fMRI assessments are needed to fully elucidate the dynamics of brain function recovery that may underlie behavioral performance improvements and clinical recovery in adolescents following concussion.

## Figures and Tables

**Table 1:** Demographics and clinical data for adolescents with concussion (i.e., concussed) and healthy control adolescents (i.e., controls).

	Concussed	Controls	Statistics
N	45	32	
Number of females (%)	16 (35%)	16 (50%)	ꭓ2 (1) = 0.4, *p* = 0.5
Race Number of Caucasian adolescents (%) Number of Black adolescents (%) Number of adolescents of unknown or more than one race (%)	38 (85%) 7 (13%) 0	34 (78%) 5 (19%) 3 (4%)	ꭓ2 (3) = 4.4, *p* = 0.2
Mean age in years (SD) [min–max]	15.9 (1.3) [13–17.9]	15.5 (1.2) [13–17.4]	t(75) = 1.3, *p* = 0.2
Mean IQ (SD) [min, max]	104.3 (8.4) [83.0–122.0]	107.9 (8.0) [92.0–124.0]	t(77) = −1.8, *p* = 0.07
Mean VOMS scores total (SD) [min, max]	43.9 (43.4) [0.0–193.0]	-	na
Mean PCSS score total (SD) [min, max]	28.9 (21.3) [0.0–79.0]	-	na
Mean number of days between injury and scan (SD) [min, max]	3.1 (2.3) [0–9]	-	na
Mean number of days between baseline scan and 6-month scan (SD) [min, max]	190.3 (13) [168–209]	-	na
Mean number of days between injury and clearance for return to play (SD) [min, max]	30.2 (33.9) [2–175]	-	na
Mean number of days between the clearance date and 6-month scan (SD) [min, max]	159.9 (40) [25–200]	-	na

## Data Availability

The data presented in this study are available on request from the corresponding author.

## Supplementary Materials

The following supporting information can be downloaded at: https://www.mdpi.com/article/10.3390/jcm13123585/s1, Figure S1: The effect of the n-back task difficulty and distractor emotion on RT, accuracy and brain activation in concussed adolescents and HCs.
