# Supplementary figures and images for: Working Memory Recovery in Adolescents with Concussion: Longitudinal fMRI Study
Source: J Clin Med. 2024 Jun 19;13(12):3585. doi: 10.3390/jcm13123585 (PMC11204632; doi:10.3390/jcm13123585)

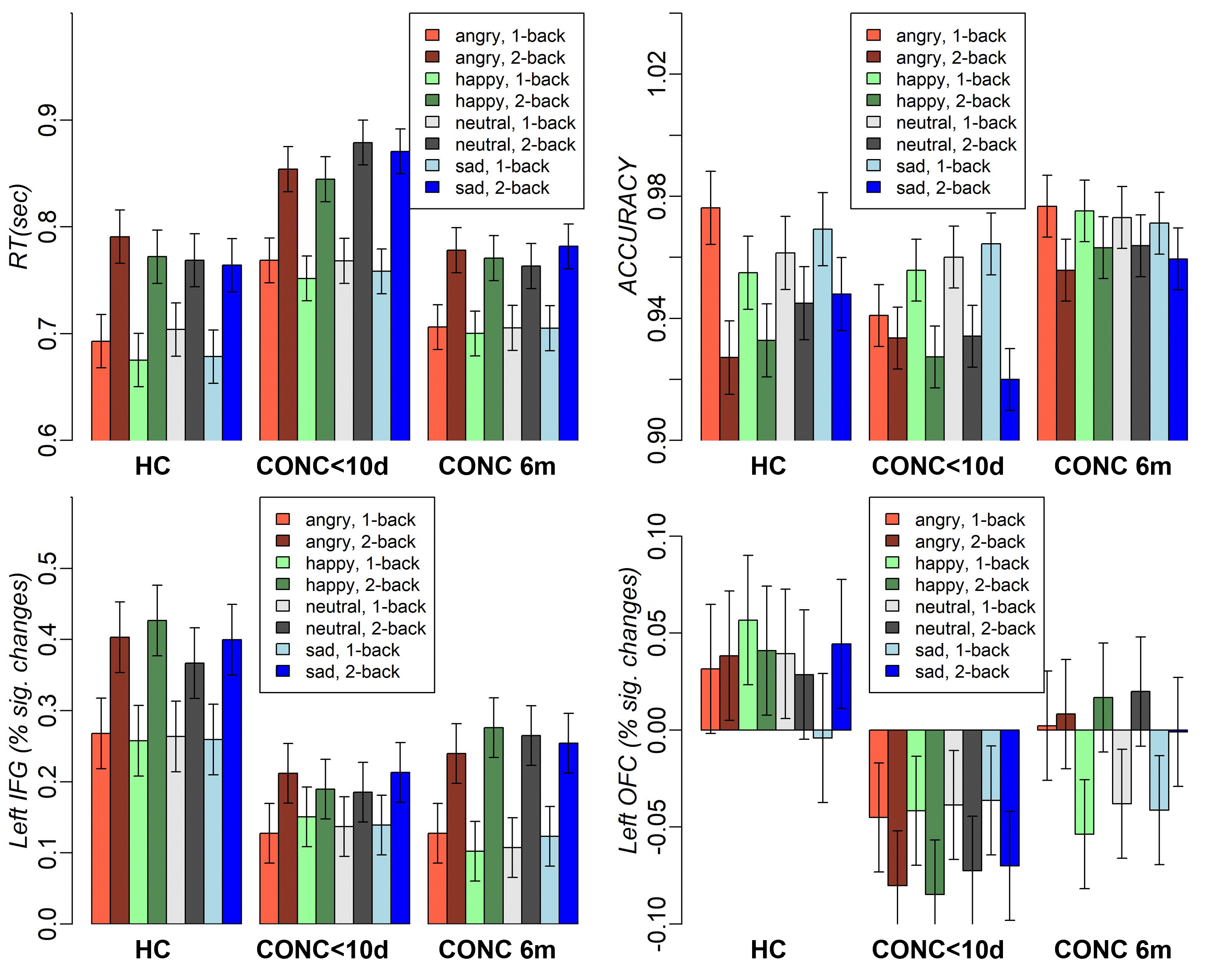

Supplement: Supplementary file 1 [file jcm-13-03585-s001.zip › jcm-3033708-supplementary.gif]
